# Stem Cells toward the Future: The Space Challenge

**DOI:** 10.3390/life4020267

**Published:** 2014-05-30

**Authors:** Silvia Bradamante, Livia Barenghi, Jeanette A.M. Maier

**Affiliations:** 1 CNR-ISTM, Institute of Molecular Science and Technologies, Via Golgi 19, 20133 Milano, Italy; 2 Integrated Orthodontic Services s.r.l., Via Cavour 52C, 23900 Lecco, Italy; E-Mail: livia.barenghi@libero.it; 3 Department Biomedical and Clinical Sciences L. Sacco, Università di Milano, Via GB Grassi 74, 20157 Milano, Italy; E-Mail: jeanette.maier@unimi.it

**Keywords:** microgravity, mechanosignaling, RPM, osteoblasts, bone, mesenchymal stem cells, spaceflight

## Abstract

Astronauts experience weightlessness-induced bone loss due to an unbalanced process of bone remodeling that involves bone mesenchymal stem cells (bMSCs), as well as osteoblasts, osteocytes, and osteoclasts. The effects of microgravity on osteo-cells have been extensively studied, but it is only recently that consideration has been given to the role of bone MSCs. These live in adult bone marrow niches, are characterized by their self-renewal and multipotent differentiation capacities, and the published data indicate that they may lead to interesting returns in the biomedical/bioengineering fields. This review describes the published findings concerning bMSCs exposed to simulated/real microgravity, mainly concentrating on how mechanosignaling, mechanotransduction and oxygen influence their proliferation, senescence and differentiation. A comprehensive understanding of bMSC behavior in microgravity and their role in preventing bone loss will be essential for entering the future age of long-lasting, manned space exploration.

## 1. Introduction

The studies of the bone loss observed in astronauts after spaceflight [1] and in ground simulated microgravity experiments (bed rest, hindlimb unloading [HLU] experiments, as well as *in vitro* studies of cellular models) have been extensively reviewed by Nagaraja and Risin [2]. Bone loss ranges from 1%–2% to 12%–24% per month in space-flown animals [3] and from 2%–9% in astronauts, with slow and often only partial recovery. This degeneration has been mainly attributed to altered bone tissue regenerative growth and repair, and a distorted responsiveness to factors present in the micro-environment: *i.e.*, reduced or absent gravitational forces decrease the integrity of osteoblasts and increase bone resorption by osteoclasts. However, it is only recently that consideration has been given to the possibility that unbalanced bone remodeling in spaceflight may be orchestrated by bone marrow mesenchymal stem cells (bMSCs) as well as osteoblasts, osteocytes and osteoclasts [4,5,6,7,8].

Mesenchymal stem cells (MSCs) represent a stem cell population present in adult tissues that can be isolated, expanded in culture, and characterized *in vitro* and *in vivo*. Their ability to self-renew, their multipotent differentiation capacity or simply their stem capability are the main features. Bone marrow mesenchymal stem cells (bMSCs) can differentiate efficiently and robustly into anchorage-dependent cells, such as osteoblasts, chondrocytes, and adipocytes [9] and also produce active substances regulating bone homeostasis [10]. In terms of bone remodeling, the key components and biological functions of bMSCs *in vivo* are in part controlled by their niches located mainly in perivascular areas of bone marrow or close to the endosteum [11,12,13,14]. 

Over the last 10 years, studies have shown that all cells can respond to applied or cell-generated mechanical forces by activating mechanosensors that mediate the complex process of biological mechanotransduction [15,16,17]. Accordingly, it is widely recognized that defects in mechanotransduction can contribute to human diseases and atypical mechanical stresses, and that the normal mechanotransduction modulate cell processes and cause tissue function impairment or failure [17,18]. The cytoskeleton, the extracellular matrix (ECM) and adhesion complexes, and membranes are the first and most common cell mechanosensors. As all proteins are deformable and therefore subject to mechanical modulation, many enzymes that change their conformation in response to force, such as kinases, phosphatases, GTPases, cyclases, and G protein-coupled receptors, create transduction pathways that lead to mechanical stress. Force transduction can also involve changes in the kinetic rate constant of a mechanosensitive enzyme or, more qualitatively, expose cryptic binding sites on a molecule [19]. The mechanotransduction mechanisms involved in bone repair and regeneration have been interpreted [20] on the basis of the tensegrity [21,22] and mechanosome theories [23]. In space research, mechanotransduction has been mainly investigated in studies of bone loss under simulated microgravity conditions, whereas most the studies of flown cells and yeast considered the cytoskeleton the main mechanosensor [24,25,26].

The aim of this review is to describe and critically examine the published data about the effects of microgravity on the characteristics and activities of bMSCs focusing on bone remodeling.

## 2. Considerations Concerning Approaches and Models

The planning and the ultimate success of a space flight experiment depends on cost-efficient studies of well-planned projects using microgravity simulators and reliable biological data derived from consolidated model systems. 

Stem cells are classically defined on the basis of their multipotency and self-renewal capacity [27], but many authors [12,28,29] have pointed out ambiguities regarding the nature, identity, function, isolation and experimental handling of MSCs. Given the ability of bMSCs to differentiate to osteoblasts, adipocytes and chondroblasts under standard *in vitro* conditions, differences in the methods used to select bMSC populations give rise to difficulties in interpreting the results. Over the years, conventional selection based on cell adherence to plastic has been improved by the use of isolation based on surface mesenchymal markers, such as STRO-1, CD146, CD105, ALP, CD49a and CD271 in human cell cultures, and nestin, CD105, VCAM1 and CD90 in mice cell cultures [9]. However, the heterogeneity of bMSC culture procedures still makes it difficult to draw any definite conclusions from the literature. 

### 2.1. Microgravity Simulators

The characteristics, configurations and limitations of the most widely used microgravity simulators (the 2-D clinostat, the random positioning machine [RPM], the rotating wall vessel [RWV] and the more recent diamagnetic levitation) have recently been reviewed. It is worth noting that the various simulators operate on the basis of different physical principles, and the experimental conditions and methods of simulating microgravity are often not equally suited for the chosen processes and organisms. Some papers provide inadequate descriptions of how the simulators were operated and/or the type of stimulation used (*i.e.*, acceleration and/or shearing forces), thus leading to confusing and/or contradictory results [30]. Furthermore, a number of national space agencies have developed their own microgravity simulators, the characteristics of which are often not completely revealed and, in today’s very active field of tissue engineering, a number of bioreactors have been developed with the aim of scaling up MSC cultures [31,32,33].

### 2.2. Experimental Model Organisms

It is not clear what model system is the most suitable for space research. As the mouse has been generally considered an ideal organism because of its striking similarity to humans and the fact that it can be used as a genetic model for understanding human biology and disease, it has also been selected for *in vitro* and *in vivo* space research. 

The bMSC cultures used in many *in vitro* experiments were not well characterized because of differences in the isolation protocols (see above), passages (the maximum lifespan of hMSCs is 41 ± 10 population doublings in the case of young donors and 24 ± 11 population doublings in the case of older donors) [34], different growth factors [35] and proliferative potential [36,37,38]. It is also necessary to reinterpret some of the data in the light of the updated characterization of some of the model cell cultures used in simulated microgravity and spaceflight experiments. For example, the cultures were cell lines rather than isolated primary bone cells [39,40,41,42,43,44] and, although they had some osteoblastic features, they were different in terms of their proliferation kinetics, osteoid production and receptor activities. 

In the case of *in vivo* experiments, difficulties arise from the poorly standardized age, sex and strain of the animals, and differences in the experimental protocols and times of exposure to microgravity [2]. 

### 2.3. Osteogenic Media

Dexamethasone (DEX) and 1α,25-dihydroxy Vitamin D3 (Vit D_3_) are the most widely used non-proteinaceous chemical compounds known to promote the osteogenic differentiation of MSCs *in vitro* [45], although DEX is generally chosen for laboratory experiments because of its rapid action. However, there are major differences between the two compounds: when used in experiments of osteogenic differentiation, Vit D_3_ is less efficient in promoting adipocyte and osteoclast differentiation. In detail, Vit D_3_ (but not DEX): (a) induces the expression of bone morphogenetic protein-2 (BMP-2) during osteoblast differentiation [46]; (b) inhibits adipocyte differentiation and gene expression in murine bMSCs [47,48]; (c) stabilizes its own receptor, Vitamin D receptor (VDR) [48]; and (d) inhibits adipogenesis in the TMS-14 line of pre-adipocytes that support osteoclast-like cell formation [49]. 

Recently, MC3T3-6OSE2-Luc cells [50], osteoblasts transfected with a vector expressing luciferase under the control of runt-related transcription factor (Runx) responsive consensus, were cultured under simulated microgravity with or without Vit D_3_. In simulated microgravity after 48 h of clinorotation, luciferase activity was lower than that at 1 g; the same trend was true also after treatment with Vit D_3_ *i.e.*, lower in simulated microgravity than at 1 g condition. Co-immunoprecipitation showed that the interaction between VDR and Runx2 was decreased by simulated microgravity. On these bases, the authors conclude that gravity affects the response of Runx2 to Vit D3. These results are in keeping with a recent report showing that a rapid decrease of bone mineral density correlated with polymorphisms for the genes of VDR and of collagen, type I, alpha 1 (Col1a1) in the majority of cosmonauts [51]. 

### 2.4. Oxygen Tension (pO_2_)

*In vivo*, human MSCs reside in specific “perivascular niches” [52] in close association with cells, blood vessels and matrix glycoproteins. This three-dimensional space provides a highly specialized microenvironment in which contact and communication are critical for MSC self-renewal and multipotency. The control of the niche depends on the dynamic equilibrium of many factors (oxygen concentration, cytokine gradients, pH, ionic and electrical potentials, available nutrients, substrate mechanics and mechanical forces) that act in temporal and spatial patterns [53]. 

Although MSCs are located close to vascular structures, the various tissues in which they are found are characterized by a low oxygen tension (pO_2_) of about 2%–8% [54] that has been interpreted as a source of selective advantages, one of the most important being the possibility of escaping the DNA damage caused by the generation of reactive oxygen species (ROS). It has been shown that pO_2_ plays a key role in regulating the fate of stem cells [54]. Under hypoxia, hypoxia-activating factors (HIFs) are stabilized and translocated to the nucleus, where they act as transcription factors for genes regulating proliferation and differentiation. Under normoxic conditions, HIFs are degraded and the genes that regulate osteogenic differentiation are promoted [33]. However, like most other cells, stem cells are typically cultured in traditional incubators in 20% O_2_, even though the variation in oxygen tension must be considered an additional variable in studies of the responses induced by microgravity [6]. 

In the niches, bMSCs are very close to the blood vessels that assist the diffusive mass transport of various compounds (particularly oxygen) in order to maintain their viability. This could explain why in rats subjected for 28 days to HLU the reduced blood flow and, consequently, bone perfusion corresponds to alterations in the balance between bone resorption and bone formation This finding is in line with those of *in vitro* studies indicating that diffusive mass transport is impaired under conditions of microgravity, thus possibly leading to deficiencies in the molecules involved in mechanosignalling and/or mechanotransduction (ions, prostaglandin E_2_, transcription factors, *etc.*) [55,56,57]. One recent study has confirmed that osteogenesis is highly dependent on oxygen supply [58].

3. bMSC Proliferation, Telomerase Activity, and Differentiation in Simulated and Real Microgravity

Cells are profoundly affected by the reduction in gravitational force. Data are sometimes conflicting, but some common denominators have been individuated, from the relevant cytoskeletal disorganization, which is implicated in mechanotransduction and affects cell signaling, proliferation, migration and death, to a massive genetic reprogramming, as an attempt to adapt to microgravity [59,60].

### 3.1. Experiments in Simulated Microgravity

Some conflicting results about the proliferative behavior of MSCs in ground-based, simulated microgravity studies can be found and should be ascribed to the differences of the experimental conditions, *i.e.*, the type of microgravity simulator used, the growth on 2D or 3D, different levels of oxygenation and different growth factors utilized. Among them, it has been reported that simulated microgravity inhibits the growth of rat bMSCs by arresting the cells in the G0/G1 phase of cell cycle and reduces the sensitivity of the cells to various growth factors [61]. It also retards their differentiation towards osteoblasts. These results were confirmed and broadened by showing that simulated microgravity forces the differentiation of rat bMSCs towards adipocytes, which are considered force-insensitive cells [62].

Analogously, simulated microgravity reduces osteoblastogenesis of human bMSCs and induces adipogenesis. Briefly, human bMSCs failed to express alkaline phosphatase (AP), collagen 1, osteonectin, and Runx2, whereas PPAR-γ2 (which is important for adipocyte differentiation), adipsin, leptin and glut-4 were all highly expressed after 7 d of simulated microgravity. The cells also showed decreased ERK and increased p38 phosphorylation, the pathways that respectively regulate the activity of Runx2 and PPAR-γ2 [4]. The reduction in osteoblastic differentiation and induction of adipocytic differentiation, initially associated with reduced integrin signaling [63] was then mainly attributed to the large increase in G-actin, reduced RhoA activity and the subsequent phosphorylation of cofilin [64]. 

Gene expression analyses have been used by other groups to evaluate the effects of different periods of simulated microgravity on human bMSCs. Briefly, 120 hours up-regulated the expression of the genes generally involved in cell adhesion, the regulation of proliferation and some signaling pathways, and down-regulated the expression of those involved in cell differentiation [65]; 20 days induced significantly altered the expression of 144 genes mainly involved in inflammatory responses, intracellular interactions, matrix and adhesion, metabolic processes, signaling and regulation, including 30 that were up-regulated, whereas the expression of many of the genes involved in osteogenic differentiation (COLI5A1, CXCL12, DPT, WISP2) or interactions between MSCs and other bone marrow cells (CXCL12; SCG2) was reduced [66]. The effects of seven days were analyzed using a whole genome microarray and gene ontology (GO) [67], and the results indicated 882 down-regulated and 505 up-regulated genes. GO clustering for molecular function revealed that most of the genes related to the cytoskeleton, nucleus and extracellular matrix (ECM) were down-regulated, whereas cell membrane protein and chromatin-related genes were up-regulated. Clustering of the genes in groups of related biological processes indicated the down-regulation of cell proliferation and apoptosis, cell adhesion, and differentiation. Clustering them into groups related to different signaling pathways showed that the genes related to cell adhesion, cell communication, cell cycle, and cytoskeleton were down-regulated, whereas those relating to cytokine/cytokine receptor interactions, MAPK cascade, the metabolism of various amino acids, and cell lineage were up-regulated. The overall conclusion was that specific genes of osteogenesis are mainly down-regulated in comparison with static cultures, whereas adipogenic differentiation benefits from microgravity. Although these experiments were performed using different microgravity simulators (desktop RPM for [65,66] and RWV for [67]) and the gene expression analyses were unrelated, the overall picture indicates that microgravity decreases osteogenesis and increases adipogenesis. 

The same conclusions were reached using HLU model, which is significantly similar to spaceflight in terms of functional and structural changes and is therefore considered a good model for *in vivo* microgravity simulation, although it is important to note that spaceflight unloads the entire body whereas HLU only unloads the hindlimbs [68]. In one experiment [69], rats had their hind limbs elevated for five days, after which their tibial bMSCs were harvested and cultured. In comparison with controls, they expressed 50% less c-*fos* mRNA and 35% less osteocalcin mRNA, and there was a significant decrease in proliferation and mineralization; there was also an apparent discrepancy between increased AP gene expression and decreased in AP enzyme activity. Experiments based on 14 days of HLU have shown that the number of femoral osteoprogenitors identified on the basis of osteoblast CFUs decreased by 71% in six-week-old rats and only 16.6% in six-month-old rats, which highlights the importance of animal selection; moreover, the proliferative capacity of osteoblastic colonies identified on the basis of colony size of young rats was reduced by 20% [70]. The overall mechanisms of MSC proliferation and osteogenesis under conditions of microgravity is still unclear; nevertheless, simulated microgravity has been successfully used in the development of tissue engineering and, particularly, in the field of bone and cartilage reconstruction, as recently summarized by Barzegari and Saei [71].

Although various lines of evidence indicate that stem cells have evolved more stringent mechanisms of genome integrity protection than differentiated and proliferating cells, it is generally agreed that telomeric DNA undergoes gradual erosion as MSCs accumulate consecutive doublings [72], and that MSCs show an age-related increase in mutation with patterns of clonal evolution toward tumor formation [73]. Yuge *et al.* [74] found that seven days of simulated microgravity did not alter the telomere length or telomerase activity of human bMSCs, but this was not confirmed when rat bMSCs were exposed to five days of simulated microgravity as there was a significant decrease in telomerase activity, decreased osteogenesis and increased adipogenesis [75]. 

### 3.2. Experiments in Space

The space flight opportunities for biomedical experiments are rare and suffer from a number of operative constraints that could bias the validity of the experiment itself, but remain a unique opportunity to confirm and explain the effects due to microgravity, that are only partially activated/detectable in simulated conditions. In our experience [76,77], many studies performed in simulated microgravity were the basis to shape up a simple, suitable, hopefully significant spaceflight experiment. At the moment, however, no clear results from experiments focussing on bMSCs in real microgravity are available. Most of the cells used until now possess a “mature” phenotype or are not fully characterized. In one such study, murine osteoclast precursors were cultured for four days in space on a synthetic three-dimensional bonelike biomaterial, whereas osteoclasts (OCs) were cultured for 10 days (OSTEO experiment. ESA FOTON M3 mission). In comparison with ground control samples, the real microgravity led to a several-fold increase in the genes involved in osteoclast maturation and activity, such as integrin β_3_, cathepsin K, MMP-9 and the calcitonin receptor [78]. During the same FOTON M3 mission, murine osteoblasts and osteoclasts were cultured with the aim of making a post-flight analysis of focal adhesion sites, microtobules, F-actin and nuclear morphology: the results indicated variations in cell shape, an increase in the diameter of intact nuclei, and significantly more disrupted and often fragmented or condensed nuclei, all of which is highly suggestive of programmed cell death [43]. 

Due to the limited spaceflight opportunities, most of the data come from animal (rat and mouse) models with the results being obtained from experiments performed at the end of the mission when the animals were sacrificed. It is worth noting that the developmental status of the animals plays an important role as space-flown old rats are less responsive to microgravity (there are fewer changes in their skeletal structures), probably due to a lower metabolic rate and increased cell aging [79], but their production of early osteoblastic progenitors and pre-osteoblastic cells is unaltered [80]. 

A recent study has compared the effects of a long 91-day stay on the ISS on wild-type and pleiotrophin-transgenic mice, but the results indicating that transgenic mice better tolerate microgravity need to be confirmed [81]. 

## 4. Mechanobiology

Physics plays a decisive role in controlling the fate of stem cells. Higuera *et al.* have recently published a review on how MSCs respond to physical forces [82]. Clarifying the mechanisms that are disturbed by reduced gravity will not only be useful to overcome spaceflight-induced alterations, but also will improve strategies for new biotechnological applications.

As has been pointed out by many authors, stem cells have structural, mechanical, and biochemical properties that are quite different from those of fully differentiated cells, such as their cytoskeletal organization and elasticity, membrane tension, cell shape, and adhesive strength. Some of these properties may play an important role in cell fate and differentiation [83]. MSCs consistently proliferate or differentiate upon cues from hydrostatic pressure, diffusive mass transport, shear stress, surface chemistry, mechanotransduction, and molecular kinetics. 

A number of recent and sophisticated studies have shown that cell adhesion, and responses to mechanical stimuli such as compressive, shear, and osmotic stresses, cyclic stretching, hydrostatic pressure and matrix stiffness influence the differentiation of MSCs into specific lineages [84]. It has also been shown that the ECM and its physical and biophysical interactions with cells can influence cell development and fate [85].

It has been reported that *in vitro* MSCs respond to a fluid flow stimulus by releasing ATP and increasing their proliferation, with the activation of MAP kinase signaling and an increase in intracellular calcium [86]. This behavior indicates that MSCs respond to biophysical signals via mechanisms similar to those of more mature cell types [87]. An updated and fascinating description of MSC mechanobiology has recently been published [88]. 

**Figure 1:**
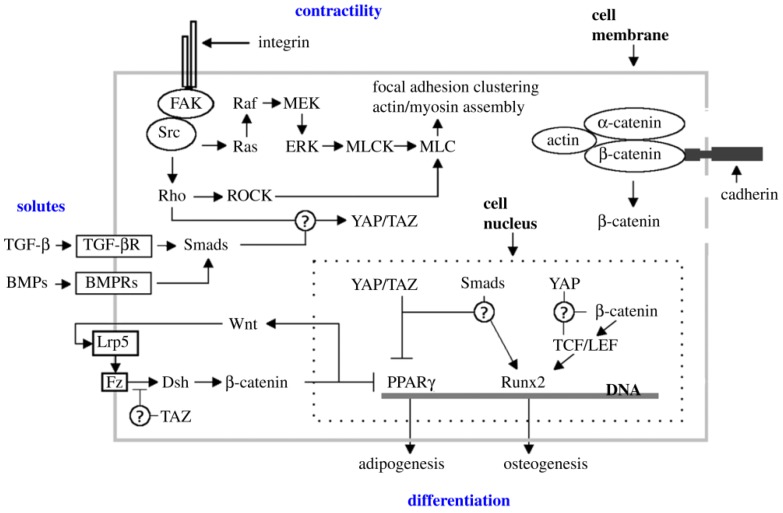
A simplified diagram of the signaling pathways involved in contractility-based mechanosensing and mesenchymal stem cell (MSC) proliferation.

Now that it seems to have been established that the shape and fate of MSCs are greatly influenced by their mechanical interactions with the micro-environment, the important thing is to elucidate how their mechanical cues are sensed and transduced into signals that will modulate gene expression. A recent interesting article suggests that the sensors and mediators of the mechanical inputs derived from the ECM are Yes-associated protein (YAP) and a transcriptional co-activator with a PDZ-binding motif (TAZ) (Figure 1) [89]. 

## 5. Conclusions

Unfortunately, the mechanisms responsible for the bone loss suffered by astronauts have not yet been identified. However, the interest in regenerative medicine, and particularly the use of stem cells, is greatly improving our understanding of the influence and importance of the environment on stem cell fate. Mechanical forces are as crucial as genes and chemical signals for controlling the embryological development, morphogenesis, and tissue patterns. Cells are sensitive to the environment to which they respond by means of mechanosensors and mechanotransduction pathways that are still not completely clear. The life, self-renewal and differentiation of MSCs are determined by many physical and chemical inputs that ultimately lead to activation of a specific transcriptional program. Although mechanobiology is a relatively young area of research, recent results explain the alterations in fundamental biological mechanisms induced by the absence of gravity, and future research will help us to interpret the interconnections between chemical processes and mechanical cues with the support of mathematical relationships provided by physics. Combining insights on MSC behavior from simulated and real microgravity can offer new perspectives thus paving the way to the exploitation of novel strategies to prevent or cure diseases on earth and in space. 

## References

[1] Orwoll E.S., Adler R.A., Amin S., Binkley N., Lewiecki E.M., Petak S.M., Shapses S.A., Sinaki M., Watts N.B., Sibonga J.D. (2013). Skeletal health in long-duration astronauts: Nature, assessment, and management recommendations from the NASA Bone Summit. J. Bone Miner. Res..

[2] Nagaraja M.P., Risin D. (2013). The current state of bone loss research: Data from spaceflight and microgravity simulators. J. Cell. Biochem..

[3] Blaber E.A., Dvorochkin N., Lee C., Alwood J.S., Yousuf R., Pianetta P., Globus R.K., Burns B.P., Almeida E.A. (2013). Microgravity induces pelvic bone loss through osteoclastic activity, osteocytic osteolysis, and osteoblastic cell cycle inhibition by CDKN1a/p21. PLoS One.

[4] Zayzafoon M., Gathings W.E., McDonald J.M. (2004). Modeled microgravity inhibits osteogenic differentiation of human mesenchymal stem cells and increases adipogenesis. Endocrinology.

[5] Monticone M., Liu Y., Pujic N., Cancedda R. (2010). Activation of nervous system development genes in bone marrow derived mesenchymal stem cells following spaceflight exposure. J. Cell. Biochem..

[6] Versari S., Klein-Nulend J., van Loon J., Bradamante S. (2013). Influence of Oxygen in the Cultivation of Human Mesenchymal Stem Cells in Simulated Microgravity: An Explorative Study. Microgravity Sci. Technol..

[7] Wang N., Wang H., Chen J., Zhang X., Xie J., Li Z., Ma J., Wang W., Wang Z. (2013). The simulated microgravity enhances multipotential differentiation capacity of bone marrow mesenchymal stem cells. Cytotechnology.

[8] Buravkova L.B., Gershovich P.M., Gershovich J.G., Grigoriev A.I. (2013). Microgravity and Mesenchymal Stem Cell Response. Curr. Biotechnol..

[9] Bianco P., Cao X., Frenette P.S., Mao J.J., Robey P.G., Simmons P.J., Wang C.Y. (2013). The meaning, the sense and the significance: Translating the science of mesenchymal stem cells into medicine. Nat. Med..

[10] Gershovich P.M., Gershovich Iu G., Buravkova L.B. (2009). Cytoskeleton structures and adhesion properties of human stromal precursors under conditions of simulated microgravity. Tsitologiia.

[11] Ehninger A., Trumpp A. (2011). The bone marrow stem cell niche grows up: Mesenchymal stem cells and macrophages move in. J. Exp. Med..

[12] Nombela-Arrieta C., Ritz J., Silberstein L.E. (2011). The elusive nature and function of mesenchymal stem cells. Nat. Rev. Mol. Cell Biol..

[13] Frenette P.S., Pinho S., Lucas D., Scheiermann C. (2013). Mesenchymal Stem Cell: Keystone of the Hematopoietic Stem Cell Niche and a Stepping-Stone for Regenerative Medicine. Annu. Rev. Immunol..

[14] Morrison S.J., Scadden D.T. (2014). The bone marrow niche for haematopoietic stem cells. Nature.

[15] DuFort C.C., Paszek M.J., Weaver V.M. (2011). Balancing forces: Architectural control of mechanotransduction. Nat. Rev. Mol. Cell Biol..

[16] Hoffman B.D., Grashoff C., Schwartz M.A. (2011). Dynamic molecular processes mediate cellular mechanotransduction. Nature.

[17] Jaalouk D.E., Lammerding J. (2009). Mechanotransduction gone awry. Nat. Rev. Mol. Cell Biol..

[18] Janmey P.A., Miller R.T. (2011). Mechanisms of mechanical signaling in development and disease. J. Cell Sci..

[19] Sukharev S., Sachs F. (2012). Molecular force transduction by ion channels: Diversity and unifying principles. J. Cell Sci..

[20] Huang C., Ogawa R. (2010). Mechanotransduction in bone repair and regeneration. FASEB J..

[21] Ingber D.E. (2003). Tensegrity II. How structural networks influence cellular information processing networks. J. Cell Sci..

[22] Ingber D.E. (2008). Tensegrity-based mechanosensing from macro to micro. Prog. Biophys. Mol. Biol..

[23] Pavalko F.M., Norvell S.M., Burr D.B., Turner C.H., Duncan R.L., Bidwell J.P. (2003). A model for mechanotransduction in bone cells: The load-bearing mechanosomes. J. Cell. Biochem..

[24] Hughes-Fulford M. (2003). Function of the cytoskeleton in gravisensing during spaceflight. Adv. Space Res..

[25] Walther I., Bechler B., Muller O., Hunzinger E., Cogoli A. (1996). Cultivation of Saccharomyces cerevisiae in a bioreactor in microgravity. J. Biotechnol..

[26] Vorselen D., Roos W.H., Mackintosh F.C., Wuite G.J., van Loon J.J. (2014). The role of the cytoskeleton in sensing changes in gravity by nonspecialized cells. FASEB J..

[27] Karp J.M., Leng Teo G.S. (2009). Mesenchymal stem cell homing: The devil is in the details. Cell Stem Cell.

[28] Bianco P. (2013). Reply to MSCs: Science and trials. Nat. Med..

[29] Alvarez C.V., Garcia-Lavandeira M., Garcia-Rendueles M.E.R., Diaz-Rodriguez E., Garcia-Rendueles A.R., Perez-Romero S., Vila T.V., Rodrigues J.S., Lear P.V., Bravo S.B. (2012). Defining stem cell types: Understanding the therapeutic potential of ESCs, ASCs, and iPS cells. J. Mol. Endocrinol..

[30] Herranz R., Anken R., Boonstra J., Braun M., Christianen P.C.M., de Geest M., Hauslage J., Hilbig R., Hill R.J.A., Lebert M. (2013). Ground-Based Facilities for Simulation of Microgravity: Organism-Specific Recommendations for Their Use, and Recommended Terminology. Astrobiology.

[31] Liu N., Zang R., Yang S.T., Li Y. (2014). Stem cell engineering in bioreactors for large-scale bioprocessing. Eng. Life Sci..

[32] Rodrigues C.A.V., Fernandes T.G., Diogo M.M., da Silva C.L., Cabral J.M.S. (2011). Stem cell cultivation in bioreactors. Biotechnol. Adv..

[33] Sart S.B., Agathos S.N., Li Y. (2014). Process Engineering of Stem Cell Metabolism for Large Scale Expansion and Differentiation in Bioreactors. Biochem. Eng. J..

[34] Stenderup K., Justesen J., Clausen C., Kassem M. (2003). Aging is associated with decreased maximal life span and accelerated senescence of bone marrow stromal cells. Bone.

[35] Arvidson K., Abdallah B.M., Applegate L.A., Baldini N., Cenni E., Gomez-Barrena E., Granchi D., Kassem M., Konttinen Y.T., Mustafa K. (2011). Bone regeneration and stem cells. J. Cell. Mol. Med..

[36] Phinney D.G., Kopen G., Isaacson R.L., Prockop D.J. (1999). Plastic adherent stromal cells from the bone marrow of commonly used strains of inbred mice: Variations in yield, growth, and differentiation. J. Cell. Biochem..

[37] Russell K.C., Phinney D.G., Lacey M.R., Barrilleaux B.L., Meyertholen K.E., O’Connor K.C. (2010). *In vitro* high-capacity assay to quantify the clonal heterogeneity in trilineage potential of mesenchymal stem cells reveals a complex hierarchy of lineage commitment. Stem Cells.

[38] Placzek M.R., Chung I.M., Macedo H.M., Ismail S., Mortera Blanco T., Lim M., Cha J.M., Fauzi I., Kang Y., Yeo D.C. (2009). Stem cell bioprocessing: Fundamentals and principles. J. R. Soc. Interface.

[39] Carmeliet G., Nys G., Bouillon R. (1997). Microgravity reduces the differentiation of human osteoblastic MG-63 cells. J. Bone Miner. Res..

[40] Carmeliet G., Nys G., Stockmans I., Bouillon R. (1998). Gene expression related to the differentiation of osteoblastic cells is altered by microgravity. Bone.

[41] Hughes-Fulford M., Gilbertson V (1999). Osteoblast fibronectin mRNA, protein synthesis, and matrix are unchanged after exposure to microgravity. FASEB J..

[42] Hughes-Fulford M., Lewis M.L. (1996). Effects of microgravity on osteoblast growth activation. Exp. Cell Res..

[43] Nabavi N., Khandani A., Camirand A., Harrison R.E. (2011). Effects of microgravity on osteoclast bone resorption and osteoblast cytoskeletal organization and adhesion. Bone.

[44] Pardo S.J., Patel M.J., Sykes M.C., Platt M.O., Boyd N.L., Sorescu G.P., Xu M., van Loon J.J.W.A., Wang M.D., Jo H. (2005). Simulated microgravity using the Random Positioning Machine inhibits differentiation and alters gene expression profiles of 2T3 preosteoblasts. Am. J. Physiol. Cell Physiol..

[45] Heng B.C., Cao T., Stanton L.W., Robson P., Olsen B. (2004). Strategies for Directing the Differentiation of Stem Cells Into the Osteogenic Lineage *in Vitro*. J. Bone Miner. Res..

[46] Yong-sheng Z., Yun-song L.I.U., Jian-guo T.A.N. (2006). Is 1,25-dihydroxyvitamin D3 an ideal substitute for dexame-thasone for inducing osteogenic differentiation of human adipose tissue-derived stromal cells *in vitro*?. Chin. Med. J..

[47] Kelly K.A., Gimble J.M. (1998). 1,25-dihydroxy vitamin D-3 inhibits adipocyte differentiation and gene expression in murine bone marrow stromal cell clones and primary cultures. Endocrinology.

[48] Kong J., Li Y.C. (2006). Molecular mechanism of 1,25-dihydroxyvitamin D3 inhibition of adipogenesis in 3T3-L1 cells. Am. J. Physiol. Endocrinol. MeTab..

[49] Sakaguchi K., Morita I., Murota S. (2000). Relationship between the ability to support differentiation of osteoclastlike cells and adipogenesis in murine stromal cells derived from bone marrow. Prostaglandins Leukot. Essent. Fat. Acids.

[50] Guo F.M., Dai Z.Q., Wu F., Liu Z.X., Tan Y.J., Wan Y.M., Shang P., Li Y.H. (2013). Gravity affects the responsiveness of Runx2 to 1, 25-dihydroxyvitamin D3 (VD3). Acta Astronaut..

[51] Oganov V.S., Baranov V.S., Kabitskaya O.E., Novikov V.E., Bakulin A.V., Moskalenko M.V., Aseev M.V., Voitulevich L.V. (2012). Analysis of polymorphism of bone metabolism genes and evaluation of the risk of osteopenia in cosmonauts. Hum. Physiol..

[52] Moore K.A., Lemischka I.R. (2006). Stem cells and their niches. Science.

[53] Badylak S.F., Nerem R.M. (2010). Progress in tissue engineering and regenerative medicine. Proc. Natl. Acad. Sci. USA.

[54] Mohyeldin A., Garzon-Muvdi T., Quinones-Hinojosa A. (2010). Oxygen in stem cell biology: A critical component of the stem cell niche. Cell Stem Cell.

[55] Klaus D.M. (2001). Clinostats and bioreactors. Gravit. Space Biol. Bull..

[56] Begley C.M., Kleis S.J. (2002). RWPV bioreactor mass transport: Earth-based and in microgravity. Biotechnol. Bioeng..

[57] Rivera-Solorio I., Kleis S.J. (2006). Model of the mass transport to the surface of animal cells cultured in a rotating bioreactor operated in micro gravity. Biotechnol. Bioeng..

[58] Benjamin S., Sheyn D., Ben-David S., Oh A., Kallai I., Li N., Gazit D., Gazit Z. (2012). Oxygenated environment enhances both stem cell survival and osteogenic differentiation. Tissue Eng. Part A.

[59] Nichols H.L., Zhang N., Wen X. (2006). Proteomics and genomics of microgravity. Physiol. Genomics.

[60] Hughes-Fulford M. (2011). To infinity … and beyond! Human spaceflight and life science. FASEB J..

[61] Dai Z.Q., Wang R., Ling S.K., Wan Y.M., Li Y.H. (2007). Simulated microgravity inhibits the proliferation and osteogenesis of rat bone marrow mesenchymal stem cells. Cell Prolif..

[62] Huang Y., Dai Z.Q., Ling S.K., Zhang H.Y., Wan Y.M., Li Y.H. (2009). Gravity, a regulation factor in the differentiation of rat bone marrow mesenchymal stem cells. J. Biomed. Sci..

[63] Meyers V.E., Zayzafoon M., Gonda S.R., Gathings W.E., McDonald J.M. (2004). Modeled microgravity disrupts collagen I/integrin signaling during osteoblastic differentiation of human mesenchymal stem cells. J. Cell. Biochem..

[64] Meyers V.E., Zayzafoon M., Douglas J.T., McDonald J.M. (2005). RhoA and cytoskeletal disruption mediate reduced osteoblastogenesis and enhanced adipogenesis of human mesenchymal stem cells in modeled microgravity. J. Bone Miner. Res..

[65] Gershovich P.M., Gershovich J.G., Zhambalova A.P., Romanov Y.A., Buravkova L.B. (2012). Cytoskeletal proteins and stem cell markers gene expression in human bone marrow mesenchymal stromal cells after different periods of simulated microgravity. Acta Astronaut..

[66] Gershovich P.M., Gershovich Y.G., Buravkova L.B. (2013). Molecular genetic features of human mesenchymal stem cells after their osteogenic differentiation under the conditions of microgravity. Hum. Physiol..

[67] Sheyn D., Pelled G., Netanely D., Domany E., Gazit D. (2003). The effect of simulated microgravity on human mesenchymal stem cells cultured in an osteogenic differentiation system: A bioinformatics study. Tissue Eng. Part A.

[68] Morey-Holton E.R., Globus R.K. (2002). Hindlimb unloading rodent model: Technical aspects. J. Appl. Physiol..

[69] Kostenuik P.J., Halloran B.P., Morey-Holton E.R., Bikle D.D. (1997). Skeletal unloading inhibits the *in vitro* proliferation and differentiation of rat osteoprogenitor cells. Am. J. Physiol..

[70] Basso N., Bellows C.G., Heersche J.N.M. (2005). Effect of simulated weightlessness on osteoprogenitor cell number and proliferation in young and adult rats. Bone.

[71] Barzegari A., Saei A.A. (2012). An update to space biomedical research: Tissue engineering in microgravity bioreactors. Bioimpacts.

[72] Ksiazek K. (2009). A comprehensive review on mesenchymal stem cell growth and senescence. Rejuvenation Res..

[73] Gunes C., Rudolph K.L. (2013). The role of telomeres in stem cells and cancer. Cell.

[74] Yuge L., Kajiume T., Tahara H., Kawahara Y., Umeda C., Yoshimoto R., Wu S.L., Yamaoka K., Asashima M., Kataoka K. (2006). Microgravity potentiates stem cell proliferation while sustaining the capability of differentiation. Stem Cells Dev..

[75] Sun L., Gan B., Fan Y., Xie T., Hu Q., Zhuang F. (2008). Simulated microgravity alters multipotential differentiation of rat mesenchymal stem cells in association with reduced telomerase activity. Acta Astronaut..

[76] Bradamante S., Villa A., Versari S., Barenghi L., Orlandi I., Vai M. (2010). Oxidative stress and alterations in actin cytoskeleton trigger glutathione efflux in Saccharomyces cerevisiae. Biochim. Biophys. Acta.

[77] Versari S., Longinotti G., Barenghi L., Maier J.A.M., Bradamante S. (2013). The challenging environment on board the International Space Station affects endothelial cell function by triggering oxidative stress through thioredoxin interacting protein overexpression: The ESA-SPHINX experiment. FASEB J..

[78] Tamma R., Colaianni G., Camerino C., di Benedetto A., Greco G., Strippoli M., Vergari R., Grano A., Mancini L. (2009). Microgravity during spaceflight directly affects *in vitro* osteoclastogenesis and bone resorption. FASEB J..

[79] Montufar-Solis D., Duke P.J., Durnova G. (1992). Spaceflight and age affect tibial epiphyseal growth plate histomorphometry. J. Appl. Physiol..

[80] Garetto L.P., Morey E.R., Durnova G.N., Kaplansky A.S., Roberts W.E. (1992). Preosteoblast production in COSMOS 2044 rats: Short-term recovery of osteogenic potential. J. Appl. Physiol..

[81] Tavella S., Ruggiu A., Giuliani A., Brun F., Canciani B., Manescu A., Marozzi K., Cilli M., Costa D., Liu Y. (2012). Bone turnover in wild type and pleiotrophin-transgenic mice housed for three months in the International Space Station (ISS). PLoS One.

[82] Higuera G.A., van Boxtel A., van Blitterswijk C.A., Moroni L. (2012). The physics of tissue formation with mesenchymal stem cells. Trends Biotechnol..

[83] Titushkin I., Cho M. (2006). Distinct membrane mechanical properties of human mesenchymal stem cells determined using laser optical tweezers. Biophys. J..

[84] Wang Y.K., Chen C.S. (2013). Cell adhesion and mechanical stimulation in the regulation of mesenchymal stem cell differentiation. J. Cell. Mol. Med..

[85] Guilak F., Cohen D.M., Estes B.T., Gimble J.M., Liedtke W., Chen C.S. (2009). Control of stem cell fate by physical interactions with the extracellular matrix. Cell Stem Cell.

[86] Riddle R.C., Taylor A.F., Genetos D.C., Donahue H.J. (2006). MAP kinase and calcium signaling mediate fluid flow-induced human mesenchymal stem cell proliferation. Am. J. Physiol. Cell Physiol..

[87] Riddle R.C., Donahue H.J. (2009). From streaming-potentials to shear stress: 25 years of bone cell mechanotransduction. J. Orthop. Res..

[88] MacQueen L., Sun Y., Simmons C.A. (2013). Mesenchymal stem cell mechanobiology and emerging experimental platforms. J. R. Soc. Interface.

[89] Halder G., Dupont S., Piccolo S. (2012). Transduction of mechanical and cytoskeletal cues by YAP and TAZ. Nat. Rev. Mol. Cell Biol..

